# Assessment of Staling Aldehydes in Lager Beer under Maritime Transport and Storage Conditions

**DOI:** 10.3390/molecules27030600

**Published:** 2022-01-18

**Authors:** Dayana Aguiar, Ana C. Pereira, José C. Marques

**Affiliations:** 1 Faculty of Exact Sciences and Engineering, University of Madeira, Campus da Penteada, 9020-105 Funchal, Portugal; dayana.aguiar@staff.uma.pt (D.A.); jose.carlos.marques@staff.uma.pt (J.C.M.); 2 ISOPlexis, Centre for Sustainable Agriculture and Food Technology, University of Madeira, Campus da Penteada, 9020-105 Funchal, Portugal; 3 Department of Chemical Engineering, Chemical Process Engineering and Forest Products Research Centre, University of Coimbra, Pólo II—Rua Sílvio Lima, 3030-790 Coimbra, Portugal; 4 Institute of Nanostructures, Nanomodelling and Nanofabrication (I3N), University of Aveiro, 3810-193 Aveiro, Portugal

**Keywords:** beer exportation, vibrations, temperature, storage time, off-flavors, bottle opening system

## Abstract

Beer flavor stability is greatly influenced by external temperature, vibrations, and longer delivery times. The present study assessed the impact of transport and storage conditions on staling aldehyde evolution in lager beers across five sample groups (fresh, transport, and storage simulation, and their controls), which differed in their bottle opening system (either crown cap or ring pull cap). Maritime transport conditions (45 days of travel, vibrations of 1.7 Hz, and warm temperatures (21–30 °C)) were simulated, together with storage time in a distributor’s warehouse (up to 75 days). The results revealed that the concentration of Strecker aldehydes increased more quickly after transport and storage simulation in beer bottles with the ring pull cap opening system, and the contents of 2-methylpropanal and 3-methylbutanal, in particular, were up to three times higher. Benzaldehyde content also increased significantly, by 33% on average, in these samples. Hexanal was only found in beers with a ring pull cap that underwent transport simulation. Further storage after transport simulation significantly reduced the content of 2-methylpropanal, 3-methylbutanal, and hexanal, by 73%, 57%, and 43%, respectively, suggesting the formation of a bound state. 5-hydroxymethylfurfural was continuously increased by 78.5% and 40.5% after the Transport and Transport & Storage simulations, respectively. Transport conditions lead to a slight increase, of 0.6 EBC units, in beer color.

## 1. Introduction

Beer is the most consumed alcoholic beverage worldwide, representing a continuously growing market, due to its global commercialization and the emergence of a wide range of styles [1]. In 2018, beer production in the European Union totaled approximately 405.94 million hectoliters, an increase of about 4.2% compared to the previous five years (389.46 million hectoliters). Regarding international trade, the European Union exported about 22% of their production, namely 88.72 million hectoliters, while imports represented lower amounts [2]. Thus, the trade in beer gives rise to an additional concern for the brewing sector: how to ensure that beer maintains as much of its freshness and pleasant sensorial features as possible until the point of consumption, following exposure to warm temperatures, vibrations, and long-distance travel.

Beer is known to be a sensitive beverage, since its physicochemical properties continuously change overtime, especially when bottled beers are stored or commercialized under inappropriate conditions. Most of the available research demonstrated that prolonged beer storage under uncooled temperatures (above 20 °C) critically affects beer flavor stability, a problem common to all beer styles [1,3,4]. This continuous loss of beer freshness during storage has been linked to several phenomena that occur in the bottle, being induced not only by the oxygen content but also by temperature and storage time. This staling process leads to a continuous loss of beer bitterness, associated with the degradation of iso-α-acids, as reported by Caballero, et al. [5]. Moreover, the appearance of haze, evolution of color, and changes in volatile composition, such as loss of esters and a simultaneous increase in staling aldehydes, are the main modifications to beer flavor stability critically reviewed and reported [4,6,7,8,9]. Additionally, Lehnhardt, et al. [10] discussed changes in beer sensorial profile during storage, namely in terms of an aged flavor. In their review, carbonyl compounds (aldehydes) were identified as the principal contributors to the appearance of off-flavors during aging, such as cardboard, caramel, sherry, bread, and ribes-like flavors.

In 2014, Janssen et al. [11] reported that the formation of beer turbidity was related to the vibration and shaking experienced during distribution and emphasized the need for investigations into the impact of transportation factors. More recently, a few studies have pointed out that the impact of vibration on flavor stability of beer undergoing transport is not negligible [11,12,13,14]. More recently, one of the principal findings was that vibrations reinforce the overall loss of beer stability, acting synergistically with temperature effects. Jaskula-Goiris et al. [12] monitored the impact of maritime transport on the chemical and sensory properties of beer. It was found that distribution conditions lead to significant color changes (an increase up to 14 EBC units), with the total concentration of staling aldehydes increasing more than two times for some compounds, whereas the content of iso-α-acids and total packaged oxygen was significantly decreased, by up to 20% and 54%, respectively. Furthermore, after transportation, beers were described as less fruity and bitter and having a characteristic cardboard and musty aroma. Similar findings were reported after further investigations on beers transported by truck, it being highlighted that the staling aldehydes 2-methylpropanal, 2-methylbutanal, *trans*-2-nonenal, and furfural are more sensitive to transport conditions [13,14].

The principal contributors to undesirable changes in beer flavor are the aldehydes from Strecker degradation, lipid oxidation, and Maillard reactions. Although their formation mechanisms have been quite well studied, their presence in packaged beer and their increase during storage or after transportation is still not well understood [6]. The increase of these compounds through de novo formation in packaged beer has been considered a misconception, due to the limited levels of their precursors and the relative low pH of the final beer. Rather, these aging indicators are produced during the brewing process and end up in the final beer, either in their free state or in a reversible form bound to an adduct. These bound-state aldehydes, known as bisulfite or cysteine adducts, are currently considered one of the primary sources of aldehydes in aged beers. Due to their non-volatile character, these bound-state aldehydes are not removed by evaporation during wort boiling or reduced during fermentation, and consequently they may be present in the final beer. In their bound-state, they are undetectable in fresh beer and the same is true for their sensory perception [7,15,16]. More recently, it has been reported that during beer aging the release of bound-state aldehydes and their de novo formation occur simultaneously, although de novo formation during natural aging is more prevalent after four months [17]. Therefore, the presence of these carbonyl compounds, either in their free or bound state form, and their precursors for de novo formation, define the aging potential of the final beer, which can easily vary according to the brewing process and raw materials applied [18].

Nevertheless, little is known about the impact of transport vibrations on these compounds, since studies are scarce regarding the influence of vibration during road or maritime transport on beer flavor stability. Therefore, the focus of this research was to study the influence of temperature, vibration, and time during maritime transportation on the aldehyde profile of bottled lager beer. Considering the prominent role of aldehyde compounds on beer flavor degradation, these were chosen to measure the impact of the previously described transport and storage variables.

## 2. Results and Discussion

### 2.1. Aldehyde Profile of Fresh Lager Beers

A total of 11 carbonyl compounds classified into Strecker degradation aldehydes (2-methylpropanal, 2-methylbutanal, 3-methylbutanal, phenylacetaldehyde, and benzaldehyde), lipid oxidation aldehydes (hexanal, heptanal, nonanal and *trans*-2-nonenal), aldehydes formed by Maillard reaction (5-Hydroximethylfurfural (5-HMF)), and one miscellaneous compound (acetaldehyde) were identified in fresh lager beers. Table 1 shows the average concentration of each staling aldehyde marker per batch.

Acetaldehyde, also known as ethanal, was the most abundant aldehyde found in the analyzed fresh beers, varying in concentration from 593 µg/L to 1265 µg/L (Table 1). During the fermentation process, yeasts produce acetaldehyde as an intermediate compound in the conversion of glucose to ethanol. Higher levels of acetaldehyde in fresh beer may indicate the presence of unideal fermentation and suggest that the expected conversion of acetaldehyde to ethanol is less than optimal. Yeast viability, elevated wort oxygen concentration, and fermentation temperatures may influence acetaldehyde accumulation in beer, compromising beer flavor. In fresh beer, the acetaldehyde concentration can vary from 600 µg/L up to 2400 µg/L [19,20].

Among the Strecker aldehydes, phenylacetaldehyde was found to be predominant, with concentrations ranging from 87.64 µg/L to 97.12 µg/L (Table 1). This compound is usually found in lower concentrations in pale lager beers, from 3.10 µg/L up to 22 µg/L [19,21]. In the five batches analyzed, the concentration of benzaldehyde ranged from 5.21 µg/L to 6.41 µg/L, values of the same order of magnitude of those found in fresh pale lagers analyzed by Malfliet et al. [21] (1.10–3.20 µg/L) and by Saison et al. [19] (average values of 1.20 µg/L). The remaining Strecker aldehydes were found at lower concentrations and far from their odor thresholds [7,22]. Regarding 2-methylpropanal and 3-methylbutanal, the concentrations varied between 2.53 and 5.60 µg/L and 3.25 and 9.49 µg/L, respectively. These values are also consistent with concentrations found in similar beers, with average values of 11.00 µg/L and 9.00 µg/L, respectively [19]. In all samples analyzed, 2-methylbutanal was not detected.

Regarding the oxidation compounds formed by lipid oxidation, hexanal was only quantified in samples from two batches (B1 and B2), presenting values of around 0.80 µg/L. Similar values were found by Malfliet et al. [21], while Techakriengkrai et al. [23] found higher values in other pale lager beer samples (1.63 µg/L). Nonanal was present in concentrations between 1.81–4.47 µg/L. Heptanal, a derivative of *trans*-2-nonenal, was always found below its corresponding limit of detection (0.16 µg/L). Regarding *trans*-2-nonenal, its concentration in all samples analyzed exceeded its flavor threshold (0.03 µg/L), ranging between 0.49 and 0.74 µg/L. This compound is responsible for the cardboard/papery flavor of aged beers [7,24,25]. Malfliet et al. [21] and Saison et al. [19] found lower concentration values in pale lager beers, 0.04 µg/L and 0.03 µg/L, respectively.

Finally, 5-HMF typically associated with bready and caramel attributes of aged beers, was the only furanic aldehyde quantified. The content of this compound surpassed its odor threshold (35.78 µg/L), varying between 1.22 mg/L and up to 1.51 mg/L [7]. The analyzed fresh lager beers had at least four-times lower levels of 5-HMF than those found by Saiso et al. [19] and Techakriengkrai et al. [23].

### 2.2. Impact of Maritime Transportation on Beer: Aldehyde Evolution

To assess the influence of maritime transportation conditions on lager beer flavor stability, the evolution of the 11 aldehydes previously identified in fresh samples was monitored across five sample groups namely: fresh samples, samples subjected to shipping conditions, samples subjected to shipping conditions and additional storage time, and control samples for the last two groups. In the following subsections, these sample groups will be denoted as follows: (i) Fresh (T0), (ii) Transport simulation, (iii) Transport & Storage simulation, (iv) Transport Control, and (v) Transport & Storage Control. Next, the impact of the conditions under study and the main findings found for each compound are discussed, according to the aldehyde family (detailed quantification of the analyzed aldehydes in each sample group can be found in Supplementary material Table S1).

#### 2.2.1. Strecker Aldehydes

The four Strecker aldehydes identified in fresh samples were also present after Transport and Transport & Storage simulations, as well as in their corresponding control samples.

**Figure 1:**
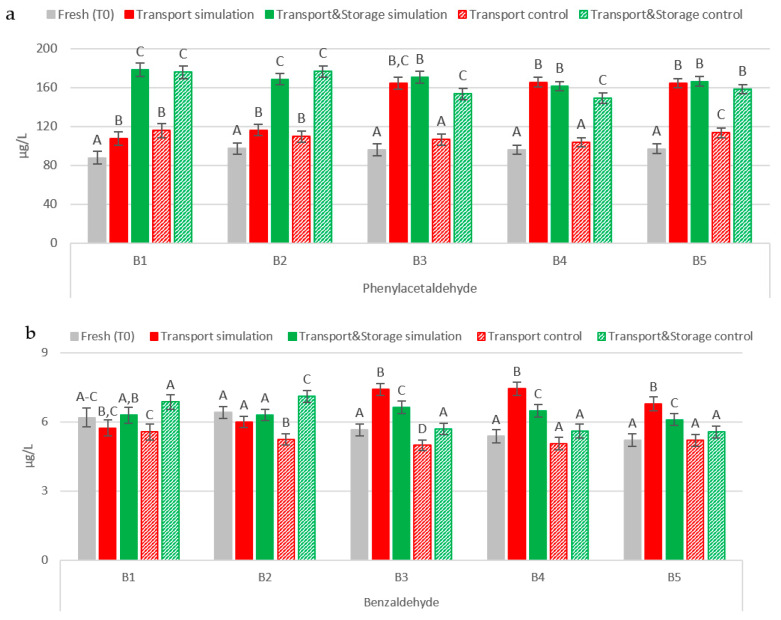
Evolution of Strecker aldehydes under simulated conditions: (**a**) Phenylacetaldehyde; (**b**) Benzaldehyde. The Tukey test was performed for each batch. Different letters represent statistically significant differences (*p* < 0.05).

Phenylacetaldehyde was the most abundant aldehyde of this family in all sample sets, with concentrations ranging between 87.64 µg/L and 178.31 µg/L (Figure 1a). It was observed that storage time at cold temperatures (2 °C) leads to significant increases (*p* < 0.01) of 21% after 45 days (B1, B2, B5) and between 55 and 101%, on average, after almost four months in the remaining batches. Higher concentrations were only observed in beers exposed to transport conditions (45 days at 21–30 °C and 1.7 Hz). Phenylacetaldehyde content continuously increased (*p* < 0.01), on average by 88% compared to fresh samples, until the end of the Transport & Storage simulation, only in the first two batches (B1 and B2). Additionally, maritime transport conditions seem to accelerate the concentration increase when the bottle opening system allows oxygen diffusion into the bottle, since a faster and accentuated increase (*p* < 0.01) of around 72% occurred in transport simulation beer bottles with a ring pull cap opening system (B3–B5). No variation was recorded after further storage of these samples. Similar trends after transport simulation have been previously reported. The experimental maritime transport simulation conducted by Jaskula-Goiris et al. [12] showed an increase of almost 50% in phenylacetaldehyde concentration (from 17.70 µg/L to 26.30 µg/L). Additionally, authors observed an increase of approximately 76% (from 10.80 µg/L up to 19.00 µg/L) in samples that underwent actual maritime transportation over a period of approximately 30 days. In addition, Paternoster et al. [13] found that phenylacetaldehyde content was two times higher (from 37.74 to 78.72 µg/L) after simulation of truck transport at 30 °C and 50 Hz, when compared to beers only kept at 30 °C. Two hypotheses can explain these results. The rate of oxidative reactions in bottled beer can be enhanced by vibration. In a previous study, vibrations were identified as the factor responsible for the absorption of headspace oxygen into the beer [13]. Thus, the reaction rate of the de novo formation of this compound can be favored but this depends on the availability of its precursor in bottled beer, since phenylacetaldehyde results from the Strecker degradation of phenylalanine. In this case, a higher diffusion of oxygen through the cap may have occurred in the last three batches, due to the ring pull cap opening system. Further incorporation into the beer was magnified by vibrations, promoting a higher rate of oxidation. Second, warm temperatures in combination with vibrations (transport conditions) and the diffusion of oxygen through the bottle cap can promote the liberation of phenylacetaldehyde from its bound state, for example into amino acids such as cysteine at a higher rate.

In the case of benzaldehyde, storage time is not critical, except for B2, where an increase of about 11% in concentration was observed in Transport & Storage control samples (Figure 1b). For this compound the transport conditions, warm temperatures and vibrations led to a significant increase (*p* < 0.01) from 5.41 µg/L up to 7.21 µg/L in beer samples with a ring pull cap opening system, although after further storage concentrations this fell to an average of 6.40 µg/L. These results differ from the values found by Jaskula-Goiris et al. [12]. In their study, no differences were reported in benzaldehyde content after maritime transport simulation. A less pronounced increase was recorded for beers that underwent truck transport simulation at 30 °C and vibrations set at 50 Hz. Benzaldehyde concentrations varied from 1.48 µg/L up to 1.70 µg/L [13]. Similarly to phenylacetaldehyde, the diffusion of oxygen through the ring pull cap opening system of these samples can catalyze the formation of these aldehydes or, in combination with the transport conditions, lead to its liberation from a bound state.

**Figure 2:**
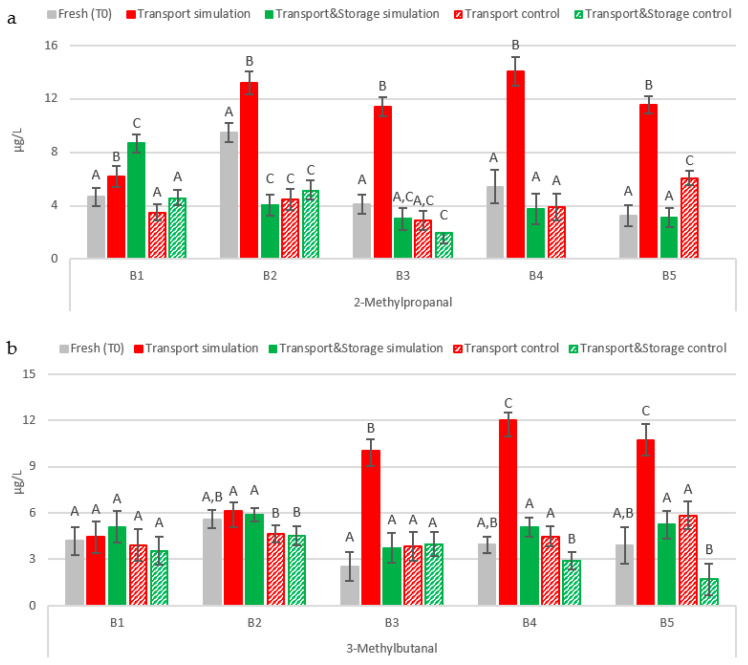
Evolution of Strecker aldehydes under simulated conditions: (**a**) 2-methylpropanal; (**b**) 3-methylbutanal. The Tukey test was performed for each batch. Different letters represent statistically significant differences (*p* < 0.05).

The behavior of 2-methylpropanal and 3-methylbutanal was similar. In general, cold storage kept the concentration of both aldehydes at similar levels in freshly packaged beer during prolonged storage. In contrast, the results from transport simulation seem to indicate that the warm temperatures and vibrations that beer faces during maritime transportation promote a significant increase in these staling markers (*p* < 0.01). In general, the concentration of 2-methylpropanal was always higher than 10 µg/L in the transport simulation samples (Figure 2a). These results are in accordance with the observed evolution in beers tested after real maritime transportation. After 51 days of maritime transport, 2-methylpropanal concentration was higher than 10 µg/L, reaching 28.5 µg/L from 4.1 µg/L in fresh beers. According to Jaskula-Goiris et al. [12], beers submitted to the maritime transport simulation doubled their initial 2-methylpropanal concentration. Paternoster et al. [13] also reported the critical effect of vibrations in combination with temperature and highlighted that the higher the exposure temperature, the greater the effect of vibrations. The authors observed an increase from 11.63 µg/L to 23.74 µg/L after a truck transport simulation at 30 °C and 50 Hz of vibrations for 90 h, whereas beers exposed to 30 °C for 90 h developed lower levels (18.83 µg/L). In contrast, the exposure to 45 °C instead of 30 °C in the transport simulation led to a total concentration of 79.64 µg/L. On the other hand, the content of 3-methylbutanal was only significantly affected on beer samples with a ring pull cap opening system, with an increase between three and four times higher after the transport simulation (Figure 2b). These results are in accordance with what was previously reported by Jaskula-Goiris et al. [12] in actually transported samples and maritime transport simulation samples. The authors quantified higher levels (6.8 µg/L) in beer shipped for 51 days, compared to 2.1 µg/L in beer kept at 0 °C. In the transport simulation, the 3-methylbutanal content was two times higher (10.3 µg/L from 5.2 µg/L). In another study, the truck transport simulation conditions also promoted an increase of this aldehyde by up to 9.90 µg/L and 10.85 µg/L from 8.84 µg/L, according to the temperature tested (30 °C and 45 °C, respectively) [13]. These differences may be related to de novo formation in bottled beer according to the availability of the precursor’s valine (2-methylpropanal) and leucine (3-methylbutanal) or by the degradation of their bound state already present in fresh beer, reactions that are enhanced by transport conditions and the diffusion of oxygen through the bottle cap, as mentioned before.

Interestingly, the content developed during the transport simulation of both aldehydes significantly decreased (*p* < 0.01) after a period of 75 days of further storage to levels similar to those found in fresh beers. In the case of 2-methylpropanal, this behavior was reported before by Paternoster et al. [13]. Beers stored at 30 °C for 60 days after the truck transport simulation (30 °C or 5 °C at 50 Hz for 90 h) had 5% and 22% less 2-methylpropanal when compared to their corresponding non-vibrated beers only kept at 30 °C and 5 °C for 90 h. These results suggest that when beers are no longer subjected to vibrations, the aldehyde can bind again to free amino acids such as cysteine, leading to a possible re-formation of a bound state, which reduces the volatility of these aldehydes, and consequently lower levels of their free state can be detected.

#### 2.2.2. Lipid Oxidation Aldehydes

**Figure 3:**
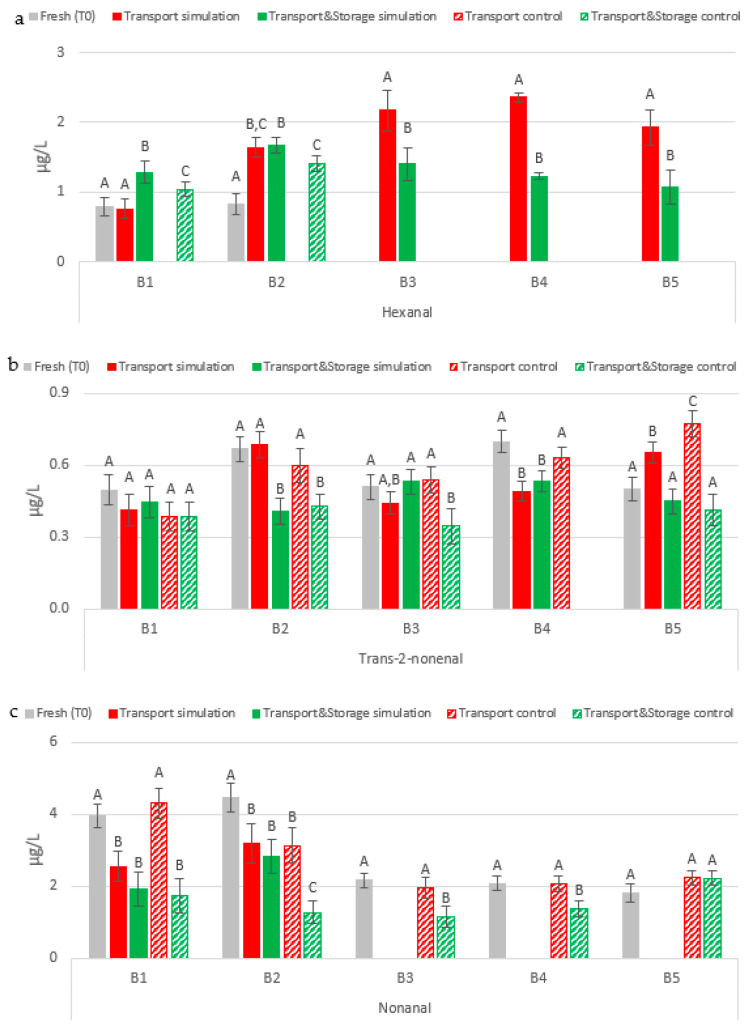
Evolution of lipid oxidation aldehydes under simulated conditions: (**a**) Hexanal; (**b**) *Trans*-2-nonenal; (**c**) Nonanal. A Tukey test was performed for each batch. Different letters represent statistically significant differences (*p* < 0.05).

Regarding lipid oxidation, both hexanal and nonanal were found to be affected by transport conditions and by the beer bottle opening system, whereas for *trans*-2-nonenal it seems that the transport conditions under study did not have any impact, since no trend in variation was observed, as represented in Figure 3.

The content of hexanal in bottled beer was not only affected by the transport conditions under study but also by the bottle opening system (Figure 3a). In beer bottles with a ring pull cap opening system (B3–B5), hexanal was only identified and quantified in the samples that underwent transport simulation for 45 days, with an average content of 2.15 µg/L. Similarly to 2-methylpropanal and 3-methylbutanal, the final content of this aldehyde in these samples significantly decreased (*p* < 0.01) by around 43% after an additional storage between 19–29 °C for 75 days. Regarding beer samples from the first two batches, only in B2 did the hexanal content double after transport simulation and no variation was observed after further storage. Only the results of the first two batches are supported by a previously reported study, where authors found that the hexanal content was duplicated after real maritime transport (from 0.4 µg/L to 0.8 µg/L), but in the performed simulation no variation in its concentration was observed [12]. In another study, truck transport simulation promoted a decrease in hexanal levels, from 0.57 µg/L to 0.43 µg/L [13]. The evolution of hexanal in the transport simulation samples of B2 to B5 suggests that vibrations, in combination with unstable temperatures, may disrupt its bound state form already present in fresh beers, resulting in higher free hexanal levels that can be detected. Moreover, the results of the transport with storage simulation for the last three batches (beers with ring pull cap opening system) indicated that the free hexanal liberated or formed during transport appeared to bind again during storage. These results are supported by a previously published study by Baert et al. [16], which reported that hexanal showed a greater tendency to be transformed into a bound state than to remain free during beer aging. The authors observed an increase of 58% in hexanal concentration after aging, whereas an increase of only 20% was detected in beers spiked with cysteine after aging, due to the strong interaction of this aldehyde with cysteine at beer pH (≈4.4).

*Trans*-2-nonenal was the first carbonyl compound linked to the appearance of cardboard off-flavor during beer aging [10]. This aldehyde was present at concentrations always higher than its flavor threshold (0.03 µg/L) [7] in all experimental sets (Figure 3b). As previously mentioned, the impact of maritime transport conditions under study on *trans*-2-nonenal levels was not clear, since this aldehyde did not show a pattern of evolution across the five experimental batches. However, similar levels to those in fresh samples were quantified after transport simulation for most batches (B1–B3), which may possibly indicate that maritime transportation is not a critical factor for this compound. These results are in accordance with the investigation by Paternoster et al. [13]. The authors did not find differences in *trans*-2-nonenal content in beers that underwent truck transport simulation at 30 °C and 50 Hz, 15 m/s^2^. In contrast, the content of this aldehyde doubles under maritime transport conditions, as demonstrated by Jaskula-Goiris et al. [12] who found that *trans*-2-nonenal levels doubled from 0.03 µg/L to 0.06 µg/L in both pilsner beers submitted to real maritime transportation or maritime transport simulation (30 days at 30 °C, 1.7 Hz and 1.14 m/s^2^). On the other hand, increases in storage time at 2 °C yielded lower or similar concentrations than in fresh beers, proving that cold storage protects bottled beer against the evolution of this staling marker.

Finally, for the first-time the evolution of nonanal during transport simulation was assessed. Nonanal had a different evolution in relation to the other identified aldehydes originated by a lipid oxidation pathway, as represented in Figure 3c. The combined effect of transport conditions, warm temperatures, and vibrations promoted a significant reduction (*p* < 0.01) of this aldehyde, by 32% on average in beer samples with a crown cap opening system (B1–B2) that underwent transport simulation. Further storage of these samples did not affect their nonanal content. In the remaining batches (beers with ring pull cap) the content of nonanal dropped to values below its limit of quantification. In terms of cold storage, similar conclusions were drawn compared to *trans*-2-nonenal. In general, beers stored at 2 °C had similar or lower levels compared to fresh samples.

#### 2.2.3. Furanic Aldehydes

**Figure 4:**
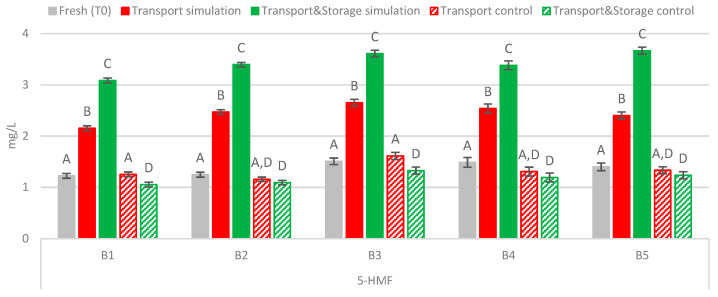
Evolution of 5-hydroxymethylfurfural under simulated conditions. A Tukey test was performed for each batch. Different letters represent statistically significant differences (*p* < 0.05).

The results in Figure 4 show that the heat-load indicator 5-HMF increased significantly (*p* < 0.01) in bottled lager beers submitted to distribution conditions in all batches analyzed, whereas in beers stored at 2 °C for up to 120 days, similar or lower levels were found. A continuous increase of this furanic aldehyde was observed across the 120 days of maritime transport simulation. The effect of temperature in combination with vibration led to a significant increase (*p* < 0.01) of 78.5% on average. Moreover, an even higher content of 5-HMF was found in transport simulation beers with additional storage, corresponding to a continuous and significant increase (*p* < 0.01) of 40.5% on average, compared to the levels developed after transport simulation. According to the limited research available on this topic, this is the first time that an impact of transport conditions on 5-HMF levels of bottled lager beer has been reported. Nevertheless, some studies found an increase of this furanic aldehyde in bottled beers under storage conditions. Techakriengkrai et al. [23] reported that 5-HMF content after 28 days of storage at 30 °C was four times higher than in fresh lager beers. An increase of around 60% was also found in lager beers stored at 28 °C for 3 months [19]. Regarding furfural, the limited research available on this topic reports that furfural tends to significantly increase during distribution, being sensitive to the action of both vibrations and temperature [12,13]. Despite this, under the transport conditions simulated in this study, furfural was not quantified in any sample.

#### 2.2.4. Acetaldehyde

**Figure 5:**
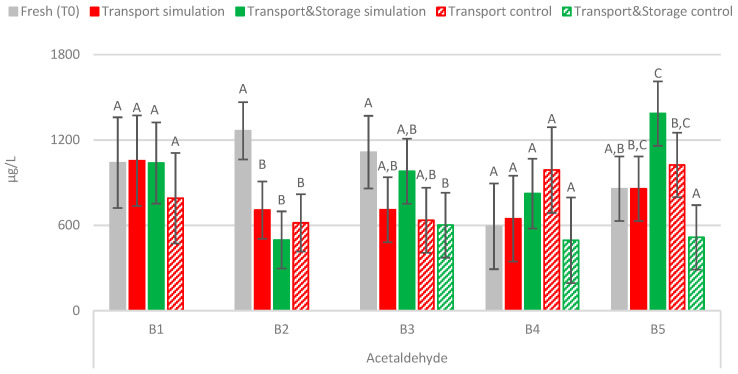
Evolution of acetaldehyde under the simulated conditions. A Tukey test was performed for each batch. Different letters represent statistically significant differences (*p* < 0.05).

For the first time, the impact of transport conditions on the acetaldehyde content of bottled lager beers was assessed (Figure 5). Similar to *trans*-2-nonenal, the combined effect of temperature with vibration did not affect, in general, the acetaldehyde concentration, since no significant variations (*p* < 0.01) were observed between the fresh samples and transport simulation or Transport & Storage simulation samples. Thus, it seems that this carbonyl compound is not sensitive to the combined effect of temperature (19–30 °C) and vibrations (1.7 Hz) during a transportation period of up to 120 days. Similar behavior was observed for the control samples during cold storage.

### 2.3. Beer Color

**Figure 6:**
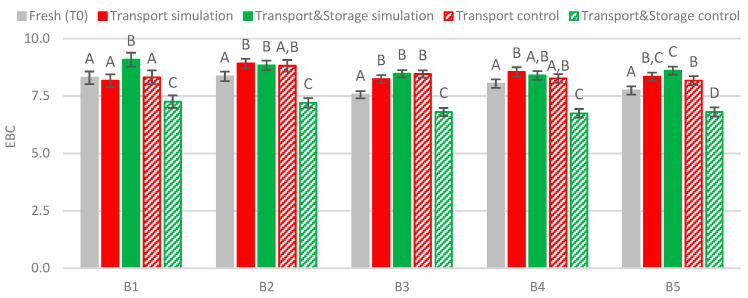
Evolution of beer color under simulated conditions. A Tukey test was performed for each batch. Different letters represent statistically significant differences (*p* < 0.05).

In general, the results show differences between fresh beers and transport simulation beers (Figure 6). The maritime transport conditions simulated (45 days of travel at 21–30 °C and 1.7 Hz) led to a slight, but significant, increase in beer color of 0.6 EBC units on average (*p* < 0.01), whereas in transported beer samples with additional storage for 75 days between 19–29 °C, no variation in color was observed when compared to their corresponding transport simulation samples. As previously reported by Jaskula-Goiris et al. [12], pilsner beers transported by ship also showed significant differences in color compared to fresh samples. Beers transported from Belgium to Japan had an increase of 0.7 EBC units, and pale beer exported to the USA had an increase of 1.7 EBC units. The highest increase found was 14 EBC units in a dark specialty beer. These color changes can result from oxidative reactions and subsequent degradation of polyphenols, due to oxygen present in bottled beer or due to melanoidins that are formed through the Maillard reaction [26,27]. In contrast, prolonged cold storage (2 °C) of up to 120 days resulted in a significant diminishment of beer color, of 1 EBC unit on average.

## 3. Materials and Methods

### 3.1. Chemicals

All chemicals used had a purity grade higher than 95%. Hexanal, benzaldehyde, 2-methylpropanal, nonanal, 2-methylbutanal, 3-methylbutanal, and 4-fluorobenzaldehyde standards were purchased from Sigma-Aldrich (Steinheim, Germany). Acetaldehyde, phenylacetaldehyde, 5-(hydroxymethyl) furfural, 2-furaldehyde and *trans*-2-nonenal were purchased from Acros Organics (Geel, Belgium). Absolute ethanol, acetonitrile, and methanol (all HPLC grade 99.99%) came from Sigma-Aldrich (Steinheim, Germany). Sodium chloride was obtained from Panreac (Barcelona, Spain). Ultra-pure water with a resistivity of >18 MΩ·cm (type 1) was obtained from a Millipore Simplicity^®^ UV apparatus (Milford, MA, USA). The alkane solution (C7–C30) was obtained from Supelco (Sigma Aldrich, St. Louis, MO, USA).

### 3.2. Beer Samples

Aldehyde compounds were determined in 120 lager beer samples kindly donated by a local brewery. This lager beer has an alcohol content of 5.1% [28]. A total of five different batches were analyzed, differing in terms of bottle opening system (crown cap and ring pull cap) and bottle volume (20 cL and 30 cL).

### 3.3. Setup of Maritime Transport and Storage Simulation Experiment

This study simulated the longest shipping route in the world (from Madeira Island to China) to study the impact of transport and storage conditions on beer flavor stability markers. In particular, the temperature, time, and vibration which beer is subjected to during maritime transport were simulated at laboratory scale. Online weather databases were consulted to determine the average temperatures for 2018 to 2020 for all the maritime transport route points [29,30]. Summer temperatures were considered (end of June to October). These monthly average temperatures were used to simulate the transportation and storage conditions. According to Jaskula-Goiris et al. [12], the vibrations that bottled beer experiences during maritime transport correspond to frequencies of 1.7 Hz (102 rpm). These vibrations and the journey temperature profile were simulated at laboratory scale, using an incubator with orbital agitation (Comecta–Ivymen, Spain).

**Figure 7:**
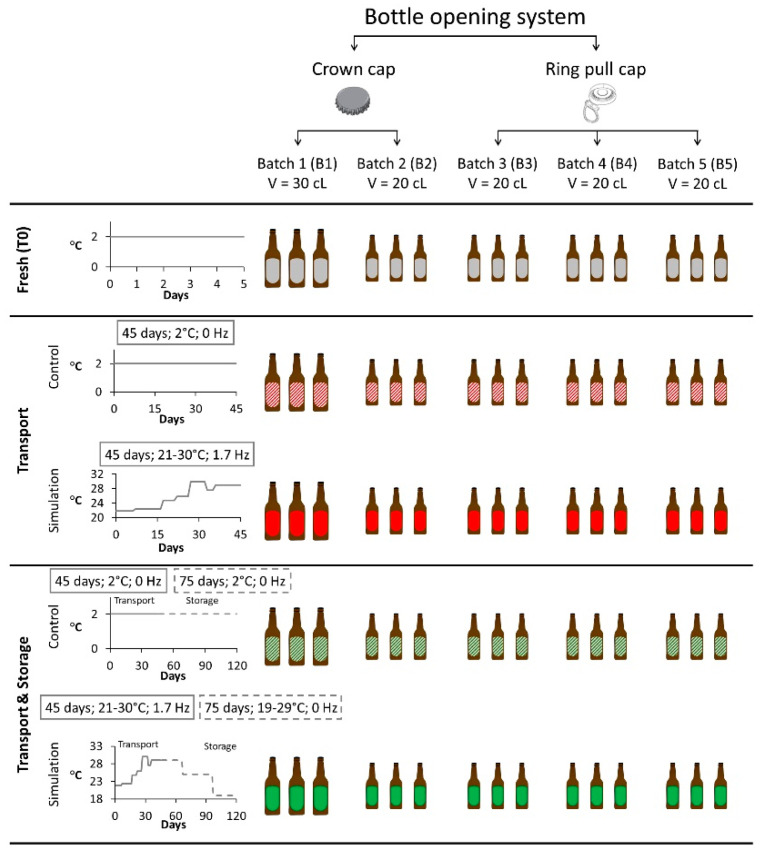
Overview of the experimental design to simulate maritime transport and storage conditions.

The commercial lager beer was exposed for a period of 45 days to a range of temperatures between 21 °C and 30 °C with vibrations set at 1.7 Hz, to simulate maritime transportation. After transport simulation, half of the samples were stored in the dark at a range of temperatures between 19 °C and 29 °C for 75 days, to simulate storage conditions upon arrival at the distributor’s warehouse. Fresh samples and control samples of each simulation group were kept at 2 °C. The five sample groups defined to evaluate the conditions previously described are given in the results and discussion section as follows: (i) Fresh (T0), (ii) Transport simulation, (iii) Transport & Storage simulation, (iv) Transport Control, and (v) Transport & Storage Control. Figure 7 summarizes the number of samples analyzed and the conditions that each group experienced.

### 3.4. Quantification of Aldehydes via HS-SPME-GC-MS

Headspace-solid phase microextraction-gas chromatography-mass spectrometry (HS-SPME-GC-MS) was carried out according to the conditions optimized and reported by Vieira et al. [31], with minor modifications. The sample preparation consisted of adding 3.3 g of sodium chloride and 10 mL of the sample in a 20 mL capped glass vial. Then 5 µL of the internal standard 4-fluorobenzaldehyde at a concentration of 50 mg/L was added. Extraction was performed using a Carboxen/Polydimethylsiloxan (CAR/PDMS) fiber coating (85 µm film thickness) for 20 min at 40 °C. In the present study, the extraction was performed in an automatic TriPlus autosampler in SPME mode. The extraction procedure was performed in triplicate for all samples under study.

Gas chromatography–mass spectrometry (GC-MS) analyses were carried out using a TRACE GC Ultra gas chromatograph coupled to an ISQ single quadrupole from Thermo Scientific (Hudson, NH, USA). The employed capillary column was a TRB-WAX column (60 m × 0.25 mm) with 0.25 µm film thickness (Teknokroma, Barcelona, Spain). Helium was employed as the carrier gas and was injected at a constant flow rate of 1 mL/min. The injector port was kept at 260 °C, in splitless mode, while the transfer line and the ion source were maintained at 240 °C. The oven temperature program started at 50 °C, was held for 2 min, initially increased up to 100 °C at 3 °C/min, then increased up to 159 °C at 6 °C/min, and finally up to 230 °C at 35 °C/min and kept at this temperature for 7 min. The total GC run time was about 40 min. The first chromatograms were obtained by spiking a mixture of standards solution in different beer samples, in order to obtain the retention time (tR) of each target aldehyde and to confirm that there were no coeluted compounds (both in full scan (total ion count) and selective ion monitoring (SIM) mode. After the confirmation of the tR, the analyses were always performed with the characteristic and major ions of each analyte and the characteristic ions were used for quantification purposes. Since, in this study, seven more aldehydes were studied in addition to the three reported by Vieira et al. [31], a validation of the method was carried out. The validation results are given in Supplementary material Table S2.

The mass spectrometer was operated in electron impact (EI) mode at 70 eV. The SIM operating mode was used with the characteristic ions for each analyte. Data were recorded and processed using the Thermo Xcalibur 2.2 software, and compound identification was performed by comparing the mass spectra with those in the NIST08 and Wiley 6.0 libraries, and by comparing the obtained Kovats indexes with those stated on NIST Chemistry WebBook. The comparison between the obtained mass spectra with those present in the MS library databases was only considered when a fair match was achieved (>80%). Quantification was achieved by external calibration.

### 3.5. Quantification of Furanic Aldehydes by HPLC

The separation and quantification of 5-HMF and furfural compounds was conducted according to Pereira et al. [32], on a Waters Alliance Liquid Chromatograph (Milford, MA, USA) equipped with an auto-injector (Waters 2695) and a photodiode array detector (Waters 2996) system. The chromatographic separation was performed using the following mobile phases: 10 mM phosphate buffer, pH 2.70 with phosphoric acid (A), acetonitrile (B), and methanol (C). The gradient program used varied from 100% aqueous mobile phase to 60% organic phase in 58 min, followed by a 12 min re-equilibration. The Atlantis^®^ T3 column (4.6 × 250 mm id; 5 µm; Milford, MA, USA) was thermostated at 30 °C, and the mobile phase was set to a flow rate of 1.0 mL/min.

The identification of the compounds was performed by analyzing the UV-Vis spectra from 200 to 400 nm and retention times and spiking samples with pure analytes. Quantification was carried out at 280 nm, according to the external standard calibration curve previously validated. Each sample was analyzed in duplicate.

### 3.6. Determination of Beer Color

Beer color was determined according to the European Brewery convention (EBC) spectrophotometric method on a dual-beam Shimadzu UV–Vis 2600 spectrophotometer (Shimadzu, Kyoto, Japan). The analysis was performed in duplicate, using quartz cuvettes with an optical thickness of 10 mm, with ultrapure water as a blank and each sample filtered. The absorbance was measured at 430 nm. Beer color, expressed in EBC units, was calculated by multiplying the absorbance value by 25.

### 3.7. Statistical Analysis

The data analysis was conducted using Minitab^®^ 17 (Minitab, LLC, State College, PA, USA), primarily using one-way ANOVA and Tukey test, with a significance level of α = 0.05. This statistical analysis was performed to compare the five sample groups for each batch, in order to determine the impact of transport and storage conditions on each of the beer staling aldehydes under study.

## 4. Conclusions

Flavor stability of bottled beer is affected, not only by inadequate storage temperatures, but also by distribution conditions, namely by the combination of warm temperature and vibration that beer experiences during distribution. The results of this study clearly demonstrate that the maritime transport conditions simulated (45 days of travel at 21–30 °C and 1.7 Hz) contribute to a flavor deterioration, due to a significant increase in levels of staling aldehydes, particularly in bottled beers with a ring pull cap opening system. In terms of Strecker aldehydes, transport and storage conditions promote a pronounced increase in these compounds, of up to four times, when beer bottles have a ring pull cap opening system. Additionally, in beers with this opening system, the content of lipid oxidation aldehyde hexanal significantly increases after transport simulation. In contrast, nonanal was only present in beers with crown cap and its content continuously decreased. The maritime transport conditions under study did not affect the concentration of *trans*-2-nonenal and acetaldehyde in bottled lager beer. Finally, 5-HMF was found to be significantly affected by transport conditions, showing an increase of 78.5% on average.

Moreover, in beer samples that were stored for 75 days at 19–29 °C after transport simulation, the developed content of Strecker aldehydes 2-methylpropanal and 3-methylbutanal and the lipid oxidation aldehyde hexanal significantly dropped, by up to 73% in beer samples with ring pull cap, due to a potential reformation of a bound-state. In contrast, the furanic aldehyde (5-HMF) continuously increased (40.5% on average) for the 120 days of transport simulation and storage.

Last, beer color suffered a slight but significant increase of 0.6 EBC units after transport simulation, remaining similar after further storage.

## Figures and Tables

**Table 1:** Concentration of aldehydes in fresh lager beers (three beer samples per batch). Values expressed as mean value ± standard deviation.

µg/L	B1	B2	B3	B4	B5
2-methylpropanal	4.67 ± 0.74	9.49 ± 1.23	4.08 ± 0.84	5.42 ± 1.24	3.25 ± 0.98
2-methylbutanal	nd	nd	nd	nd	nd
3-methylbutanal	4.18 ± 1.09	5.60 ± 0.84	2.53 ± 0.60	3.86 ± 0.56	3.11 ± 1.61
Benzaldehyde	6.35 ± 0.75	6.41 ± 0.54	5.65 ± 0.47	5.37 ± 0.39	5.21 ± 0.23
Phenylacetaldehyde	87.64 ± 6.56	97.12 ± 7.16	95.92 ± 7.71	96.12 ± 6.57	96.96 ± 8.33
Hexanal	0.80 ± 0.24	0.84 ± 0.10	nd	nd	nd
Heptanal	nd	nd	nd	nd	nd
Nonanal	3.96 ± 0.50	4.47 ± 0.65	2.17 ± 0.25	2.09 ± 0.21	1.81 ± 0.50
*Trans*-2-nonenal	0.50 ± 0.10	0.67 ± 0.07	0.52 ± 0.10	0.74 ± 0.11	0.49 ± 0.09
Acetaldehyde	1040.70 ± 134.14	1265.00 ± 280.91	1114.60 ± 193.55	593.00 ± 292.50	858.00 ± 250.27
5-HMF (mg/L)	1.22 ± 0.03	1.25 ± 0.03	1.51 ± 0.04	1.49 ± 0.02	1.40 ± 0.05

nd, not detected.

## Data Availability

Not applicable.

## Supplementary Materials

The following are available online, Table S1: Detailed quantification results of the 11 aldehydes (three samples per batch) in fresh, control, and maritime transport simulation samples. Table S2: Performance results for the HS-SPME-GC-MS implemented methodology to quantify aldehydes in lager beer.
