# Supplementary material for: Assessment of Staling Aldehydes in Lager Beer under Maritime Transport and Storage Conditions
Source: Molecules. 2022 Jan 18;27(3):600. doi: 10.3390/molecules27030600 (PMC8839358; doi:10.3390/molecules27030600)
Supplement: Supplementary file 1 [file molecules-27-00600-s001.zip › Table S1.pdf]

Supplementary material

Table S1: Detailed quantification results of the 11 aldehydes (three samples per batch; mean ± standard deviation) in fresh, controls and maritime transport simulation samples.

Compound	Batch	Fresh (T0)	Transport simulation	Transport&Storage simulation	Transport simulation control	Transport&Storage simulation control
			**Strecker Degradation Aldehydes**			
Phenylacetaldehyde (µg/L)	1	87.64±6.56^{A}	107.16±10.54^{B}	178.31±8.13^{C}	115.55±5.47^{B}	175.89±14.87^{C}
	2	97.12±7.16^{A}	116.14±8.96^{B}	168.46±7.91^{C}	109.64±10.47^{B}	176.43±8.71^{C}
	3	95.92±7.71^{A}	164.52±8.38^{B,C}	170.38±11.20^{B}	106.46±3.74^{A}	153.37±10.81^{C}
	4	96.12±6.57^{A}	165.60±7.91^{B}	161.28±7.74^{B}	103.52±5.78^{A}	149.22±6.77^{C}
	5	96.96±8.33^{A}	164.62±4.44^{B}	166.21±9.37^{B}	113.41±6.81^{C}	158.23±5.74^{B}
Benzaldehyde (µg/L)	1	6.19±0.75^{A,B,C}	5.74±0.32^{B,C}	6.29±0.31^{A,B}	5.57±0.35^{C}	6.86±0.68^{A}
	2	6.41±0.54^{A}	5.99±0.21^{A}	6.30±0.33^{A}	5.23±0.35^{B}	7.10±0.37^{C}
	3	5.65±0.47^{A}	7.41±0.21^{B}	6.64±0.52^{C}	4.98±0.25^{D}	5.69±0.24^{A}
	4	5.37±0.39^{A}	7.44±0.42^{B}	6.47±0.32^{C}	5.06±0.35^{A}	5.60±0.57^{A}
	5	5.21±0.23^{A}	6.79±0.47^{B}	6.10±0.58^{C}	5.19±0.20^{A}	5.56±0.27^{A}
2-methylpropanal (µg/L)	1	4.67±0.74^{A}	6.18±0.15^{B}	8.67±1.38^{C}	3.50±0.48^{A}	4.55±0.63^{A}
	2	9.49±1.23^{A}	13.22±1.06^{B}	4.03±0.58^{C}	4.48±0.56^{C}	5.13±0.64^{C}
	3	4.08±0.84^{A}	11.43±1.07^{B}	3.01±0.57^{A,C}	2.88±0.44^{A,C}	2.00±0.68^{C}
	4	5.42±1.24^{A}	14.07±2.28^{B}	3.74±0.41^{A}	3.91±0.63^{A}	nd
	5	3.25±0.98^{A}	11.54±0.58^{B}	3.21±0.41^{A}	6.07±0.90^{C}	nd
2-methylbutanal (µg/L)	1	nd	nd	nd	nd	nd
	2	nd	nd	nd	nd	nd
	3	nd	nd	nd	nd	nd
	4	nd	nd	nd	nd	nd
	5	nd	nd	nd	nd	nd
3-methylbutanal (µg/L)	1	4.18±1.09^{A}	4.45±1.43^{A}	5.10±1.15^{A}	3.91±0.24^{A}	3.56±0.59^{A}
	2	5.60±0.84^{A,B}	6.12±0.29^{A}	5.89±0.45^{A}	4.64±0.74^{B}	4.51±0.71^{B}
	3	2.53±0.60^{A}	10.06±1.08^{B}	3.74±0.59^{A}	3.83±1.16^{A}	3.98±0.80^{A}
	4	3.95±0.56^{A,B}	12.01±1.05^{C}	5.07±0.52^{A}	4.48±0.53^{A}	2.91±0.41^{B}
	5	3.90±1.61^{A,B}	10.74±1.82^{C}	5.24±0.67^{A}	5.84±0.82^{A}	1.70±0.32^{B}
			**Lipid Oxidation Aldehydes**			
Hexanal (µg/L)	1	0.80±0.24^{A}	0.77±0.05^{A}	1.29±0.17^{B}	nq	1.04±0.11^{C}
	2	0.84±0.10^{A}	1.64±0.17^{B,C}	1.67±0.18^{B}	nq	1.42±0.19^{C}
	3	nd	2.17±0.42^{A}	1.41±0.15^{B}	nq	nd
	4	nd	2.36±0.02^{A}	1.24±0.07^{B}	nq	nd
	5	nd	1.93±0.31^{A}	1.08±0.15^{B}	nq	nd
Heptanal (µg/L)	1	nd	nd	nd	nd	nd
	2	nd	nd	nd	nd	nd
	3	nd	nd	nd	nd	nd
	4	nd	nd	nd	nd	nd
	5	nd	nd	nd	nd	nd
Nonanal (µg/L)	1	3.96±0.50^{A}	2.56±0.13^{B}	1.92±0.43^{B}	4.31±0.64^{A}	1.74±0.38^{B}
	2	4.47±0.65^{A}	3.19±0.30^{B}	2.83±0.32^{B}	3.13±0.54^{B}	1.36±0.27^{C}
	3	2.17±0.25^{A}	nq	nq	1.96±0.33^{A}	1.29±0.31^{B}
	4	2.09±0.21^{A}	nq	nq	2.07±0.33^{A}	1.38±0.31^{B}
	5	1.81±0.50^{A}	nq	nq	2.25±0.31^{A}	2.23±0.17^{A}
Trans-2-nonenal (µg/L)	1	0.50±0.10^{A}	0.42±0.08^{A}	0.45±0.14^{A}	0.39±0.05^{A}	0.39±0.05^{A}
	2	0.67±0.07^{A}	0.69±0.13^{A}	0.41±0.06^{B}	0.60±0.26^{A}	0.43±0.05^{B}
	3	0.52±0.10^{A}	0.45±0.07^{A,B}	0.54±0.06^{A}	0.55±0.09^{A}	0.35±0.02^{B}
	4	0.70±0.11^{A}	0.50±0.06^{B}	0.54±0.05^{B}	0.64±0.08^{A}	nq
	5	0.51±0.09^{A}	0.66±0.08^{B}	0.45±0.04^{A}	0.78±0.08^{C}	0.42±0.09^{A}
			**Miscellaneous Aldehydes**			
Acetaldehyde (µg/L)	1	1040.70±134.14^{A}	1055.00±428.84^{A}	1038.00±130.21^{A}	791.00±391.17^{A}	nd
	2	1265.00±280.91^{A}	707.00±88.45^{B}	497.10±172.01^{B}	617.41±141.43^{B}	nq
	3	1114.60±193.55^{A}	710.00±311.84^{A,B}	980.30±205.20^{A,B}	635.50±165.19^{A,B}	602.00±298.37^{B}
	4	593.00±292.50^{A}	647.00±284.10^{A}	823.30±138.20^{A}	989.00±483.29^{A}	495.20±174.53^{A}
	5	858.00±250.27^{A,B}	857.35±267.85^{B,C}	1385.90±188.02^{C}	1024.20±169.94^{B,C}	515.80±166.58^{A}
			**Furanic Aldehydes**			
5-hydroxymethylfurfural (mg/L)	1	1.22±0.03^{A}	2.15±0.07^{B}	3.09±0.08^{C}	1.25±0.04^{A}	1.05±0.03^{D}
	2	1.25±0.03^{A}	2.47±0.03^{B}	3.40±0.05^{C}	1.16±0.05^{A,D}	1.09±0.07^{D}
	3	1.51±0.04^{A}	2.65±0.08^{B}	3.61±0.09^{C}	1.61±0.08^{A}	1.33±0.08^{D}
	4	1.49±0.02^{A}	2.54±0.05^{B}	3.38±0.20^{C}	1.31±0.07^{A,D}	1.19±0.06^{D}

	5	1.40±0.05^{A}	2.40±0.11^{B}	3.67±0.10^{C}	1.33±0.04^{A,D}	1.24±0.08^{D}
Furfurall (µg/L)	1	nd	nd	nd	nd	nd
	2	nd	nd	nd	nd	nd
	3	nd	nd	nd	nd	nd
	4	nd	nd	nd	nd	nd
	5	nd	nd	nd	nd	nd

nd - not detected; nq - not quantified
