# Supplementary material for: Assessment of Staling Aldehydes in Lager Beer under Maritime Transport and Storage Conditions
Source: Molecules. 2022 Jan 18;27(3):600. doi: 10.3390/molecules27030600 (PMC8839358; doi:10.3390/molecules27030600)
Supplement: Supplementary file 1 [file molecules-27-00600-s001.zip › Table S2.pdf]

Supplementary material

Table S2: Performance results for the HS-SPME-GC-MS implemented methodology to quantify aldehydes in lager beer.

t_R (minutes)	Analyte	Kovats index	Identification/ quantification ion (in bold)	Concentration range in literature	Linear range	R^2	LOD	LOQ	Recovery (%)			Intra-day precision (%)			Inter-day precision (%)		
									C1	C2	C3	C1	C2	C3	C1	C2	C3
**4.60**	Acetaldehyde (µg/L)	630	41, 42, **43**, 44	0.6-40 [1-4]	503.22 – 15061.3	0.999986	73.03	243.43	102.58	87.64	91.27	3.54	2.98	4.33	6.47	11.15	7.03
**5.32**	2-Methylpropanal (µg/L)	671	41, 42, 43, **72**	1-229 [1,4-9]	1.00-100.17	0.999998	0.17	0.57	87.92	99.38	97.49	2.12	6.52	3.07	20.89	5.42	18.18
**6.50**	2-Methylbutanal (µg/L)	724	**57**, 58, 86	0.7-60.41 [4,6,8,9]	1.00-60.16	0.999994	0.21	0.71	104.25	98.87	91.67	4.48	5.80	6.17	11.43	12.09	15.59
**6.60**	3-Methylbutanal (µg/L)	727	44, **58**, 71, 86	0.97-57.20 [2,4,6,8-10]	1.00-60.10	0.999995	0.19	0.62	93.35	99.41	98.39	6.55	1.17	6.49	12.50	9.25	10.79
**10.45**	Hexanal (µg/L)	1064	44, 56, **57**, 82	0.5-36.01 [2,4,8-10]	1.00-100.08	0.999999	0.16	0.52	82.67	95.90	99.25	1.47	6.11	4.04	13.11	6.81	7.43
**22.26**	Nonanal (µg/L)	1390	56, **57**, 70, 98	1.63-24.08 [2,11,12]	2.00-30.07	0.999880	0.41	1.35	72.86	84.01	98.94	3.96	5.68	5.48	17.92	17.78	11.24
**26.40**	Benzaldehyde (µg/L)	1520	77, 105, **106**	0.5-30.9 [4,6,8,9,11]	4.01-30.04	0.999072	1.01	3.38	115.79	112.9	124.96	1.59	2.33	3.94	5.06	13.41	6.20
**26.62**	*Trans*-2-nonenal (µg /L)	1536	70, **83**, 84	0.03-20.08 [1,4,8,10]	0.05-8.00	0.999930	0.09	0.31	95.91	103.56	108.40	6.80	3.47	5.44	9.03	16.13	12.14
**29.28**	Phenylacetaldehyde (µg /L)	1638	**91**, 92, 120	3.01-132 [2,4,5,8,9]	5.01-150.29	0.999979	0.99	3.31	89.74	92.69	95.31	2.88	2.66	2.49	10.86	14.87	10.70

References

1. Vieira, A.C.; Pereira, A.C.; Marques, J.C.; Reis, M.S. Multi-target optimization of solid phase microextraction to analyse key flavour compounds in wort and beer. *Food Chem.* **2020**, *317*, 126466.
2. Vanderhaegen, B.; Neven, H.; Coghe, S.; Verstrepen, K.J.; Verachtert, H.; Derdelinckx, G. Evolution of Chemical and Sensory Properties during Aging of Top-Fermented Beer. *J. Agric. Food Chem.* **2003**, *51*, 6782-6790.
3. Gagula, G.; Mastanjević, K.; Mastanjević, K.; Krstanović, V.; Horvat, D.; Magdić, D. The influence of packaging material on volatile compounds of pale lager beer. *Food Packag. Shelf Life* **2020**, *24*, 100496.
4. Saison, D.; Vanbeneden, N.; De Schutter, D.; Daenen, L.; Mertens, T.; Delvaux, F.; Delvaux, F. Characterisation of the flavour and the chemical composition of lager beer after ageing in varying conditions. *Brew. Sci* **2010**, *63*, 41-53.
5. Saison, D.; De Schutter, D.P.; Delvaux, F.; Delvaux, F.R. Determination of carbonyl compounds in beer by derivatisation and headspace solid-phase microextraction in combination with gas chromatography and mass spectrometry. *J. Chromatogr. A* **2009**, *1216*, 5061-5068.
6. Andrés-Iglesias, C.; Nešpor, J.; Karabín, M.; Montero, O.; Blanco, C.A.; Dostálek, P. Comparison of carbonyl profiles from Czech and Spanish lagers: Traditional and modern technology. *LWT - Food Sci Technol* **2016**, *66*, 390-397.
7. Saison, D.; De Schutter, D.P.; Delvaux, F.; Delvaux, F.R. Optimisation of a complete method for the analysis of volatiles involved in the flavour stability of beer by solid-phase microextraction in combination with gas chromatography and mass spectrometry. *J. Chromatogr. A* **2008**, *1190*, 342-349.
8. Jaskula-Goiris, B.; De Causmaecker, B.; De Rouck, G.; Aerts, G.; Paternoster, A.; Braet, J.; De Cooman, L. Influence of transport and storage conditions on beer quality and flavour stability. *J. Inst. Brew.* **2019**, *125*, 60-68.
9. Malfliet, S.; Van Opstaele, F.; De Clippeleer, J.; Syryn, E.; Goiris, K.; De Coornan, L.; Aerts, G. Flavour instability of pale lager beers: Determination of analytical markers in relation to sensory ageing. *J. Inst. Brew.* **2008**, *114*, 180-192.
10. Filipowska, W.; Jaskula-Goiris, B.; Ditrych, M.; Schlich, J.; De Rouck, G.; Aerts, G.; De Cooman, L. Determination of optimal sample preparation for aldehyde extraction from pale malts and their quantification via headspace solid-phase microextraction followed by gas chromatography and mass spectrometry. *J. Chromatogr. A* **2020**, *1612*, 460647.
11. Moreira, N.; Meireles, S.; Brandão, T.; de Pinho, P.G. Optimization of the HS-SPME–GC–IT/MS method using a central composite design for volatile carbonyl compounds determination in beers. *Talanta* **2013**, *117*, 523-531.
12. Jeleñ, H.H.; Dabrowska, A.; Klensporf, D.; Nawrocki, J.; Wąsowicz, E.J.C.a. Determination of C3-C10 alifatic aldehydes using PFBHA derivatization and solid phase microextraction (SPME). Application to the analysis of beer. *Chem. Anal.* **2004**, *49*, 869.
